# ONCOR: design of the Dutch cardio-oncology registry

**DOI:** 10.1007/s12471-020-01517-8

**Published:** 2020-11-17

**Authors:** J. A. M. Kamphuis, M. Linschoten, M. J. Cramer, F. Alsemgeest, D. J. W. van Kessel, K. Urgel, M. C. Post, O. C. Manintveld, H. C. Hassing, C. Liesting, A. J. Wardeh, E. G. M. Olde Bijvank, J. Schaap, A. M. Stevense-den Boer, P. A. Doevendans, F. W. Asselbergs, A. J. Teske

**Affiliations:** 1 Department of Cardiology, Division of Heart and Lungs, University Medical Centre Utrecht, University of Utrecht, Utrecht, The Netherlands; 2 Department of Cardiology, St Jansdal Hospital, Harderwijk, The Netherlands; 3 Department of Cardiology, St Antonius Hospital, Nieuwegein, The Netherlands; 4 Department of Cardiology, Erasmus Medical Center, Rotterdam, The Netherlands; 5 Department of Cardiology, Albert Schweitzer Hospital, Dordrecht, The Netherlands; 6 Department of Cardiology, Haaglanden Medical Center, The Hague, The Netherlands; 7 Department of Cardiology, Amphia Hospital, Breda, The Netherlands; 8 Department of Internal Medicine, Amphia Hospital, Breda, The Netherlands; 9 Central Military Hospital, Utrecht, The Netherlands; 10 Netherlands Heart Institute, Utrecht, The Netherlands; 11 Health Data Research UK, Institute of Health Informatics and Institute of Cardiovascular Science, Faculty of Population Health Sciences, University College London, London, UK

**Keywords:** Cardio-oncology, Registries, Research design

## Abstract

**Background:**

The relative new subspecialty ‘cardio-oncology’ was established to meet the growing demand for an interdisciplinary approach to the management of cancer therapy–related cardiovascular adverse events. In recent years, specialised cardio-oncology services have been implemented worldwide, which all strive to improve the cardiovascular health of cancer patients. However, limited data are currently available on the outcomes and experiences of these specialised services, and optimal strategies for cardio-oncological care have not been established.

**Aim:**

The ONCOR registry has been created for prospective data collection and evaluation of cardio-oncological care in daily practice.

**Methods:**

Dutch hospitals using a standardised cardio-oncology care pathway are included in this national, multicentre, observational cohort study. All patients visiting these cardio-oncology services are eligible for study inclusion. Data collection at baseline consists of the (planned) cancer treatment and the cardiovascular risk profile, which are used to estimate the cardiotoxic risk. Information regarding invasive and noninvasive tests is collected during the time patients receive cardio-oncological care. Outcome data consist of the incidence of cardiovascular complications and major adverse cardiac events, and the impact of these events on the oncological treatment.

**Discussion:**

Outcomes of the ONCOR registry may aid in gaining more insight into the incidence of cancer therapy–related cardiovascular complications. The registry facilitates research on mechanisms of cardiovascular complications and on diagnostic, prognostic and therapeutic strategies. In addition, it provides a platform for future (interventional) studies. Centres with cardio-oncology services that are interested in contributing to the ONCOR registry are hereby invited to participate.

**Electronic supplementary material:**

The online version of this article (10.1007/s12471-020-01517-8) contains supplementary material, which is available to authorized users.

## Introduction

Improvements in the early detection and treatment of cancer over the last decades have led to increasing numbers of cancer survivors worldwide [1, 2]. Furthermore, the prognosis of patients treated in a palliative setting continues to improve and several malignancies are evolving into chronic conditions. With these advances, the prevention and management of short- and long-term treatment-related side effects are gaining importance.

Cardiovascular toxicities are among the most frequent unintended side effects of cancer treatment and can manifest themselves in many forms, including cancer therapy–related cardiac dysfunction (CTRCD), arrhythmias, and valvular, pericardial and coronary artery disease [3, 4]. Compared with the general population, cancer survivors are at increased risk of all these cardiovascular diseases [5], and have a 2–6 times higher risk of cardiovascular mortality [6]. With the growing awareness of these complications among healthcare professionals treating cancer patients and survivors and a demand for an interdisciplinary approach to the management of cancer therapy–related cardiovascular adverse events, a new discipline termed ‘cardio-oncology’ has emerged in the late ’90s [7]. The overarching aim of this subspecialty is the optimisation of cardiovascular health of cancer patients with pre-existent cardiovascular disease to enable the initiation of the antineoplastic treatment with the most optimal benefit-risk ratio, to improve cancer treatment tolerability, and to manage and to prevent cardiovascular complications.

Over the years, the focus of the field of cardio-oncology has shifted from treatment of cardiovascular complications towards preventive strategies and early detection of these complications by implementation of cardio-oncology services across many centres across the world [8, 9]. In line with these developments, the European Society of Cardiology (ESC) has released a position paper on cancer treatments and cardiovascular toxicity in 2016, which provides an overview of cardiovascular monitoring and decision-making in patients undergoing potentially cardiotoxic cancer treatment [4]. However, evidence-based clinical guidelines on cardio-oncological care are currently lacking and the recommendations in the position paper are all based on expert opinion.

Although all cardio-oncology services strive to improve cardiovascular health of cancer patients, different clinical approaches are being used according to local need, and limited data are available on the outcomes and experiences of these specialised services [10, 11]. Therefore, optimal strategies for cardio-oncological care are still unknown. Across the Netherlands, several cardio-oncology services have been established that provide care according to a standardised care pathway [12]. To gain insight into the outcomes of patients visiting these specialised services, we set up a national registry that collects data on daily practice cardio-oncological care.

## Methods

### Study design and setting

In April 2015, a standardised cardio-oncology care pathway was developed through a collaboration between the Departments of Cardiology, Haematology, Oncology and Radiology at the University Medical Centre Utrecht in Utrecht, the Netherlands [12]. Various other Dutch hospitals have adopted this care pathway and set up cardio-oncology services in recent years as well, resulting in a large number of patients receiving standardised care across the Netherlands. The ONCOR registry is a national, multicentre, observational cohort study, which is conducted at hospitals that provide care according to this pathway [12]. The study is performed in collaboration with the Netherlands Heart Institute in Utrecht, the Netherlands.

### Study population

All adult patients who currently receive or have received anticancer treatment and who have visited a cardio-oncology service, are eligible to participate. Written information about the ONCOR registry is provided by their treating physician at this service. Patients interested to participate are requested to provide written informed consent for the use of clinical data. Additionally, patients are asked for permission to be approached for future (interventional) studies.

The patient population seen at the cardio-oncology services largely consists of the following three subgroups: (1) patients monitored for cardiovascular complications whilst undergoing treatment with potential cardiotoxic agents; (2) patients screened for long-term cardiovascular complications; and (3) patients with cardiac complaints possibly related to their oncological treatment. For the first group, the focus lies on the early detection of CTRCD in patients deemed to be at (high) risk for this complication.

**Fig. 1:**
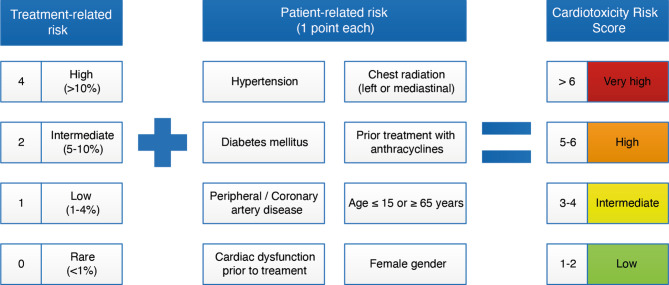
Cardiotoxicity risk score, adapted from [13]. The highest treatment-related risk score (e.g. 4, 2, 1 or 0) is used to calculate the score. For risk scores of individual anticancer agents and regimens, see ‘Medication risk score’ in Electronic Supplementary Material

The cardiotoxicity risk score [13] is utilised for CTRCD risk stratification. The cardiotoxicity risk score is based on treatment-related risk factors, e.g. use of antineoplastic agents and exposure to chest irradiation, and patient-related risk factors, such as age, sex and presence of cardiovascular risk factors and diseases ([13]; Fig. 1). Based on the estimated incidence of CTRCD associated with the use of various antineoplastic agents, the treatment-related risk is subdivided into high (>10%), intermediate (5–10%), low (1–4%), and rare (<1%) (Fig. 1; see also ‘Medication risk score’ in Electronic Supplementary Material). Currently, patients with a cardiotoxicity risk score ≥4 have an indication for referral to the cardio-oncology outpatient service to receive serial echocardiographic assessments during cancer treatment. A cut-off value of 4 was chosen in order to allocate resources to those patients who are most likely to benefit from cardio-oncological care. There are currently no risk stratification tools available for other cardiovascular complications, and referral is at the discretion of the treating oncologist.

Patients who have been treated with anthracyclines and/or mediastinal radiotherapy, are screened for late cardiotoxic effects. For example, patients with Hodgkin lymphoma or non-Hodgkin lymphoma are monitored according to the BETER protocol [14]. This protocol recommends cardiac screening every 5 years for patients treated with an equivalent dose of doxorubicin ≥300 mg/m^2^ and for those treated with an equivalent doxorubicin dose <300 mg/m^2^ in combination with mediastinal radiotherapy. Adult survivors of paediatric malignancies do not receive standard care at the cardio-oncology service, but undergo cardiovascular screening according to the LATER protocol of the Dutch Childhood Oncology Group [15]. Patients with abnormal cardiovascular findings during the LATER screening are referred to the cardio-oncology service for further management.

### Ethics and dissemination

The registry follows the Code of Conduct for the Use of Data in Health Research. Inclusion of patients is exempt from the Medical Research Involving Human Subjects Act (*WMO*) as per the judgement of the Medical Ethics Committee (METC 18-639/C, Utrecht, the Netherlands). Participating centres require approval from their local institutional ethics committee. In addition, a consortium agreement form needs to be signed, which includes specifications on obligations, liability, confidentiality and data protection. The ONCOR registry is registered at the Netherlands Trial Registry, project number NL8064 (www.trialregister.nl).

### Objectives of the ONCOR registry

**Fig. 2:**
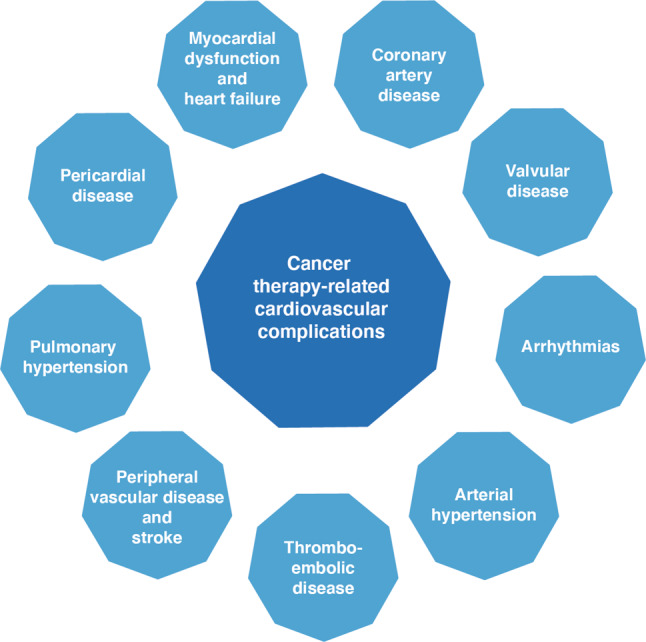
Cancer therapy – related cardiovascular complications

The objectives of the ONCOR registry are fourfold:

- To register the incidence of cardiovascular complications classified according to the ESC Position Paper on cancer treatments and cardiovascular toxicity ([4]; Fig. 2).
- To register the incidence of major adverse cardiovascular events, defined as: (i) cardiac hospitalisation (categorised by heart failure, acute coronary syndrome, arrhythmia or ‘other’); (ii) cardiac death; (iii) implantation of an implantable cardiac defibrillator or cardiac resynchronization therapy devices; or (iv) (need for) cardiac mechanical support or orthotopic heart transplantation.
- To facilitate research on mechanisms of cardiovascular complications, risk factors, and diagnostic, prognostic and therapeutic strategies.
- To provide a future platform for registry-based randomised controlled trials.

### Data collection and management

**Fig. 3:**
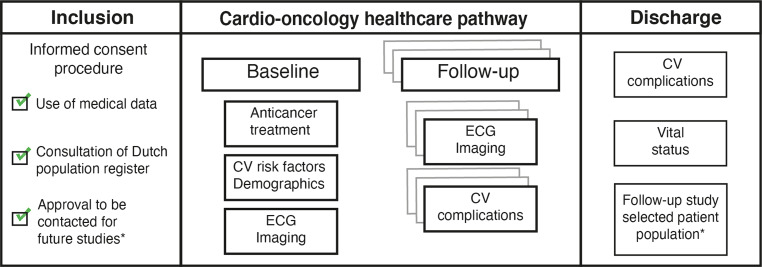
Informed consent procedure and data collection. (*CV* cardiovascular, *ECG* electrocardiography. *In case patients agree, they can be approached for future studies)

Standardised data collection instruments are used to register information collected during routine care (Fig. 3). Uniform data collection is achieved by using a standard operating procedure handbook, which provides definitions of medical terms and instructions on how questions should be answered. For parties interested in participating in the ONCOR registry, the data collection instruments, data dictionary and standard operating procedure handbook are available upon request. All data are handled confidentially and in accordance with Dutch privacy laws. Data are collected in an online REDCap database [16], which handles an audit trail that registers data access, entries and changes. The database is hosted by the Netherlands Heart Institute’s Durrer Center, which provides IT support, security and data protection. Entry of study data is performed by healthcare professionals involved in the cardio-oncology services at the participating centres. In each centre, a local coordinator is responsible for supervision of data entry. The data of each participating centre are stored in a separate data access group, which is only accessible to study members working at that centre and the national study coordinators.

### Data sharing

Local centres are free to use data from their own centre for research purposes. When involved researchers want to use data from multiple participating centres, a data request must be submitted to the ONCOR Steering Committee. This committee is made up of the local coordinators from the participating centres. For each data request, local coordinators can decide on whether data from their centre may be used.

### Timeline

The care pathway provides a standardised follow-up schedule for patients who are undergoing cancer treatment with potential cardiotoxic agents and who are monitored for CTRCD. Typically, the follow-up period for patients with active treatment is up to 1 year after completion of treatment with potentially (highly) cardiotoxic agents, as CTRCD can be detected within this time frame in a majority of patients susceptible to develop this complication [17]. The frequency of follow-up visits is dependent on the patient-specific risk score and therefore varies between patients. Depending on the clinical course, the follow-up intensity and duration can be adjusted at the discretion of the treating physician. When patients are discharged from the cardio-oncology service, the incidence of cardiovascular events is derived from the electronic health record. Information regarding follow-up will be requested in case patients receive further care in another centre. The vital status is verified in the electronic health record or in the Dutch population register in case patients are lost to follow-up.

### Baseline assessment and clinical visits during follow‑up

The data collection at baseline consists of information on the (planned) oncological treatment, including type of regimen, number of cycles, cumulative anthracycline dose and radiotherapy plans. Additionally, cardiovascular risk factors are registered. With these variables, the cardiotoxicity risk score is automatically calculated in REDCap. During the first visit and the standardised follow-up visits, clinical information on the presence of cardiac complaints and outcomes of physical examination, as well as results from electrocardiography, echocardiography and cardiac MRI, may be registered.

### Echocardiography and other cardiovascular investigations

Serial cardiac assessment is done by using echocardiography, since this imaging technique is considered to be the most feasible imaging modality in cardio-oncological practice [18]. Echocardiographic examinations are performed according to a standard protocol (see Electronic Supplementary Material). The echocardiographic assessment includes analysis of cardiac dimensions and function (including deformation imaging). Left ventricular ejection fraction measurements are preferably 3D measurements or, if not available, by the 2D biplane (modified Simpson’s) algorithm or an estimation of the left ventricular function. Valvular analysis is performed at baseline and during long-term follow-up after thoracic radiotherapy.

During follow-up, in the first year after therapy, an analysis of the valves is performed on indication (e.g. in case of a heart murmur) or when abnormalities were detected with echocardiography at baseline. All echocardiographic outcomes, as well as the name of the vendor of the ultrasound machine, can be registered in REDCap. Apart from the investigations that are part of the standardised cardio-oncology follow-up, other additional investigations are performed on indication, at the discretion of the treating physician. Outcomes of these tests, such as analysis of coronary artery disease, Holter analysis, genetic counselling and laboratory analysis, can be registered in the database. Since the value of and the optimal timing for measurement of biomarkers have not yet been established, cardiac biomarkers, such as troponin and (N-terminal prohormone of) brain natriuretic peptide, are only measured at the discretion of the treating physician; they are not yet part of the cardio-oncology care pathway [12, 19].

### Cardiovascular complications

During each clinical visit, the incidence of cardiovascular complications is evaluated. Nine predefined categories of cardiovascular complications are used to register side effects in a uniform way (Fig. 2). If a complication is diagnosed, information regarding the modalities and tests upon which the diagnosis is based, is requested in REDCap. Furthermore, the impact of the cardiovascular complication on oncological treatment is registered.

## First results

As of 12 February 2020, the ONCOR registry contains 1142 individual patient records (Tab. 1). Patients with breast cancer (39%) and haematological malignancies (50%) represent the largest patient group. Cardiac follow-up after initiation of cancer treatment is available for 1038 patients, with a median follow-up time of 21.6 months (interquartile range 10.2–60.3). During this time period, 378 cardiovascular complications have been registered, of which CTRCD was the most common (*n* = 206).

**Table 1:** First results of the ONCOR registry

Variable	Patients (*n* = 1142)
*Demographics*	
Age at treatment, years	50.8 ± 16.8
Age at first consultation, years	54.6 ± 14.2
Number of females	700 (61%)
*Malignancy*	
Breast cancer	445 (39%)
Lymphoma	214 (19%)
Acute leukaemia	177 (16%)
Haemato-oncology (other)	176 (15%)
Other	130 (11%)
*Referral reason*	
Screening during treatment	761 (67%)
Screening after treatment	184 (16%)
Cardiac complaints	197 (17%)
*Cancer therapy-related cardiovascular complications*	
Myocardial dysfunction and heart failure	206
Coronary artery disease (including coronary vasospasms)	27
Valvular disease	22
Arrhythmias	74
Arterial hypertension	7
Thrombo-embolic disease	4
Peripheral vascular disease and stroke	2
Pulmonary hypertension	2
Pericardial disease	34

Values are reported as mean ± standard deviation, *n* (%) or *n*

## Discussion

Cardio-oncology is a subspecialty in its infancy and, currently, there is a gap in knowledge regarding the added value of cardio-oncology services. One of the largest challenges that lies ahead of healthcare professionals active in this field is the identification of patients who will benefit from referral to these services prior to the initiation of cancer treatment, considering the large number of cancer patients and the accompanying restraints of resources at most cardiology departments. For each individual patient, the risk of cardiovascular complications must be weighed against the oncological prognosis. This requires close collaboration between cardiologists, oncologists and haemato-oncologists.

Patient groups likely to benefit most from active monitoring have a moderate to good oncological prognosis with a high lifetime risk of developing cardiovascular complications, such as patients with Hodgkin lymphoma, non-Hodgkin lymphoma or breast cancer [5, 6]. On the long term, with the establishment of the ONCOR registry, we hope to contribute to gaining more insight into the incidence of cardiovascular complications and the impact of early detection and treatment of cardiovascular complications on patient prognosis.

### Limitations

One of the limitations of the ONCOR registry is that the present care pathway uses a risk stratification tool to preselect patients who are currently believed to benefit from cardiac monitoring for CTRCD. This risk score predominantly depends on the cardiotoxic risk of the administered anticancer agent. Due to gaps in the existing literature concerning the cardiotoxic profile of several agents [20], this can result in the underestimation or overestimation of the true risk. In addition, the risk score has not yet been prospectively validated and introduces selection bias. Patients who are currently deemed to have a low or negligible risk of CTRCD according to this risk score, may develop cardiovascular complications that remain undetected in the subclinical phase in the absence of screening. Moreover, risk factors for the development of cardiovascular complications other than CTRCD are largely unknown.

Even though the cardio-oncological care is standardised as much as possible, the collected data originate from daily clinical practice in multiple centres; this could create issues such as vendor dependency and interobserver and intraobserver variability. These issues will be taken into account when the echocardiographic data are analysed for multicentre studies.

### Future perspectives

The current care pathway is predominantly focused on monitoring patients treated with anthracyclines and/or trastuzumab. However, the number of targeted therapies is rapidly expanding and many of these therapies are associated with specific cardiovascular complications, including myocarditis [21, 22]. The need for management of targeted therapy–related cardiovascular complications will increase and, therefore, implementation of management strategies in the care pathway is required. Additionally, the currently used risk stratification of anticancer agents can be revised when new insights into cardiotoxic profiles are available.

Expansion of the ONCOR registry with, for example, biobanks can aid in the identification of biomarkers and genetic factors and, thereby, contribute to enhancing the etiologic, diagnostic and prognostic knowledge of unintended cardiovascular effects of anticancer treatment. The establishment of an imaging biobank can help to correct issues such as interobserver and intraobserver variability. Blinded images can be examined by an independent cardiologist, using vendor-neutral analysis software. Furthermore, the registry can be used for pragmatic trials to determine optimal screening methods and therapeutic strategies [23].

## Conclusion

The ONCOR registry offers a platform for the collection of data from cardio-oncological care provided according to a standardised care pathway in various Dutch hospitals. The registry can aid in improving cardiac surveillance strategies and, thereby, contribute to the improvement of cardiovascular health in the growing population of cancer patients and survivors. Centres with a cardio-oncology service, or centres aiming to launch such a service in the future, that are interested in contributing to the ONCOR registry are hereby invited to participate.

### Caption Electronic Supplementary Material

Medication risk scores

Standard echocardiography protocol

## Appendix

### Collaborators of the ONCOR registry in the Netherlands

- Netherlands Heart Institute Cardio-oncology working group: Department of Cardiology, University Medical Center Groningen: R.A. de Boer and P. van der Meer; Department of Cardiology, Leiden University Medical Centre: M.L. Antoni; Department of Cardiology, Division of Heart and Lungs, University Medical Centre Utrecht: L.W. van Laake.
- University Medical Centre Utrecht: Department of Haematology, Cancer Centre: A. van Rhenen and R. de Weijer; Department of Medical Oncology: R.M. Bijlsma, E.H. Gort and E. van der Wall.
- St Jansdal Hospital, Harderwijk: Department of Internal Medicine: F.N. Croles (haematology) and R.J. van den Brink-Schimmel (oncology).
- St Antonius, Nieuwegein: Department of Internal Medicine: H.R. Koene (haematology); Department of Medical Oncology: M.J. Agterof, P.C. de Jong and M.J. Verhaar-Langereis.
- Erasmus Medical Center Rotterdam: Department of Hematology, Erasmus MC Cancer Institute: N. Wlazlo; Department of Medical Oncology: M.E.M.M. Bos.
- Albert Schweitzer Hospital, Dordrecht: Department of Internal Medicine: M.D. Levin (haematology).
