# Supplementary material for: ONCOR: design of the Dutch cardio-oncology registry
Source: Neth Heart J. 2020 Nov 17;29(5):288–94. doi: 10.1007/s12471-020-01517-8 (PMC8062648; doi:10.1007/s12471-020-01517-8)
Supplement: Supplementary file 2 — Standard echocardiography protocol [file 12471_2020_1517_MOESM2_ESM.pdf]

BASELINE ECHOCARDIOGRAPHY CARDIO-ONCOLOGY ONCOR

A baseline echocardiogram is performed during the 1st visit at the cardio-oncology outpatient clinic (preferably prior to initiation of cancer therapy).

Please take care of an adequate ECG signal. Each frame should include at least three heart cycles.

Report

- Dimensions (left ventricle, left atrium, proximal aorta)
- Left ventricular ejection fraction (3D)
- Global longitudinal strain
- Diastolic function
- Right ventricular dimension and function
- Right ventricular systolic pressure
- Valve analyses
- Other remarks

FOLLOW-UP ECHOCARDIOGRAPHY CARDIO-ONCOLOGY ONCOR

Follow-up echocardiographic examination will be performed upon indication, with intervals of 3 or 6 months in between.

The follow-up examination is relatively short, and focuses on the left ventricular function (systolic and diastolic). Please take care of an adequate ECG signal. Each frame should include at least three heart cycles.

Report

- Left ventricular ejection fraction (3D)
- Global longitudinal strain
- Diastolic function
- Right ventricular function
- Right ventricular systolic pressure

Imaging protocol baseline echo (1 t/m 33)

Parasternal long axis
- 2D (1)
- Color Doppler mitral- and aortic valve (2)

Parasternal short axis
- 2D Apical (3)
- 2D papillary muscles (4)
- 2D mitral valve (please note: through plane motion) (5)
- 2D aortic valve (6)
- Color Doppler aortic- (7), pulmonary- (8), tricuspid- and mitral valve
- Pulsed Wave pulmonary valve (9)

Apical 4 chamber view
- 2D (10) + focused view LV (16)
- Color Doppler mitral valve (11)
- Pulsed wave mitral valve inflow (12)
- Tissue Doppler Imaging mitral annulus (medial (13) en lateral (14))
- Pulsed wave pulmonary vein (15)

Apical 5 chamber view
- 2D (17)
- Pulsed wave left ventricular outflow tract (18)
- Continuous wave aortic valve (19)

Apical 4 chamber view - focus right ventricle
- 2D (20)
- Color Doppler tricuspid valve (21)
- Continuous wave tricuspid regurgitation (if present) (22)
- Tissue Doppler Imaging tricuspid annulus (S') (23)
- Tricuspid Annular Plane Systolic Excursion (24)

Apical 2 chamber view
- 2D (25) + focused view LV (26)
- Color Doppler mitral valve (27)

Apical 3 chamber view
- 2D (28) + focused view LV (29)
- Color Doppler aortic valve and mitral valve (30)
- Pulsed wave left ventricular outflow tract and continuous wave aortic valve

Subcostal
- Inferior vena cava (31)

3D view: full volume Left ventricle (>25Hz)(32)
Global longitudinal strain (33)

Imaging protocol follow up (red)

Parasternal long axis
- 2D (1)

Parasternal short axis
- 2D Apical (3)
- 2D papillary muscles (4)
- 2D mitral valve (please note: through plane motion) (5)
- 2D aortic valve (6)

Apical 4 chamber view
- 2D (10) + focused view LV (16)
- Pulsed wave mitral valve inflow (12)
- Tissue Doppler Imaging mitral annulus (medial (13) en lateral (14))
- Pulsed wave pulmonary vein (15)

Apical 5 chamber view
- Pulsed wave left ventricular outflow tract (18)

Apical 4 chamber view - focus right ventricle
- Continuous wave tricuspid regurgitation (if present) (22)
- Tissue Doppler Imaging tricuspid annulus (S') (23)
- Tricuspid Annular Plane Systolic Excursion (24)

Apical 2 chamber view
- 2D (25) + focused view LV (26)

Apical 3 chamber view
- 2D (28) + focused view LV (29)

3D view: full volume Left ventricle (>25Hz)(32)
Global longitudinal strain (33)

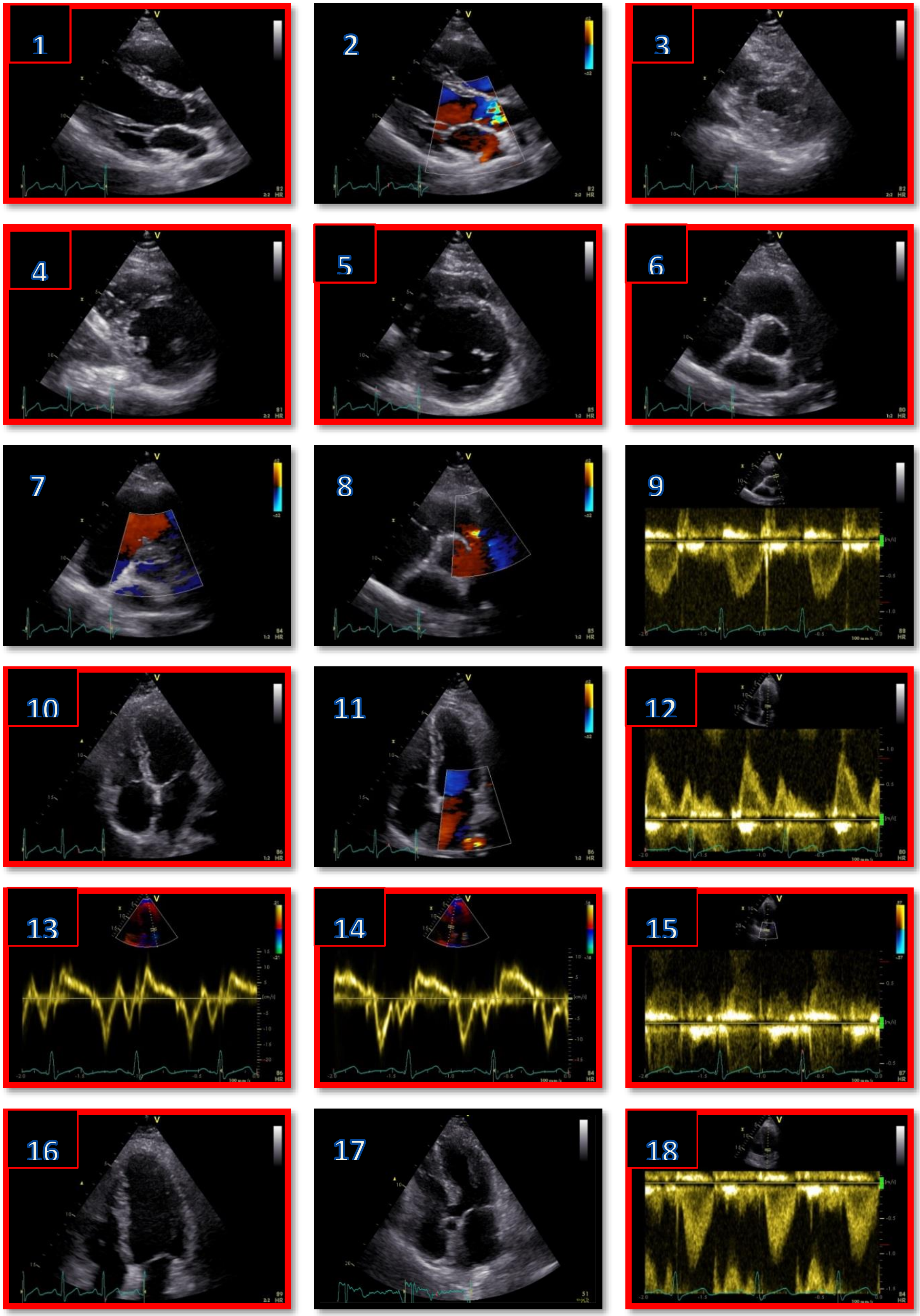
1
2
3
4
5
6
7
8
9
10
11
12
13
14
15
16
17
18

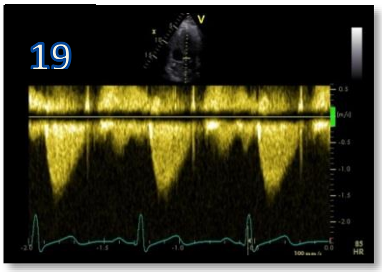
19

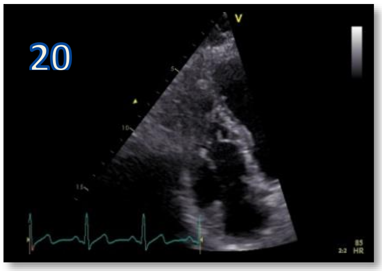
20

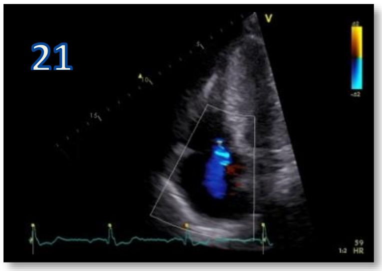
21

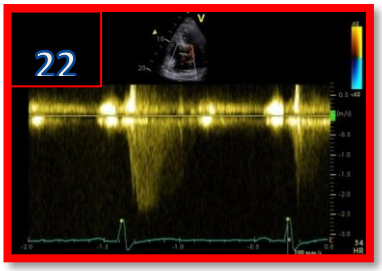
22

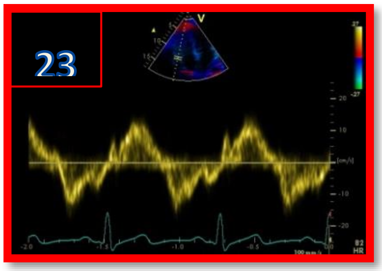
23

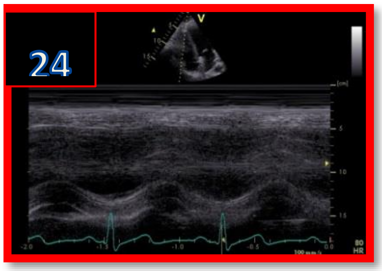
24

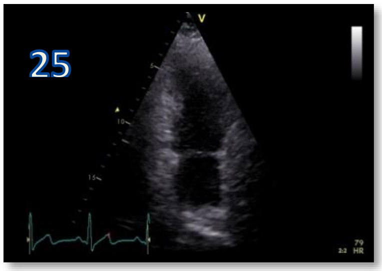
25

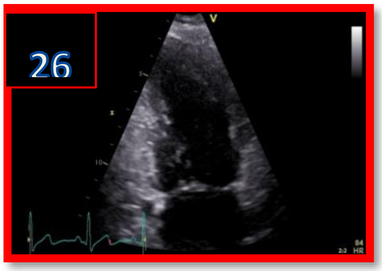
26

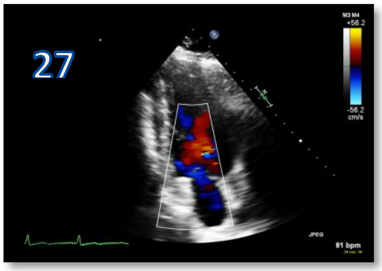
27

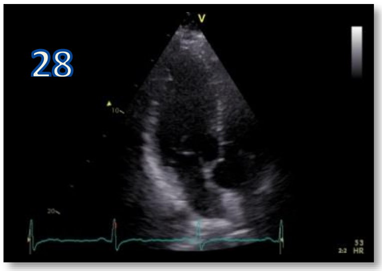
28

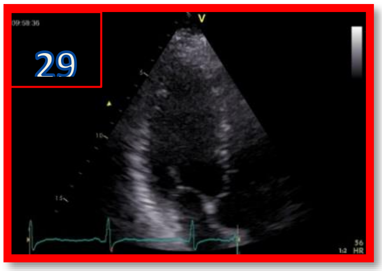
29

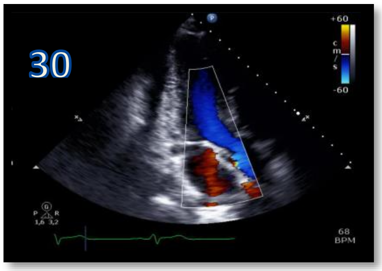
30

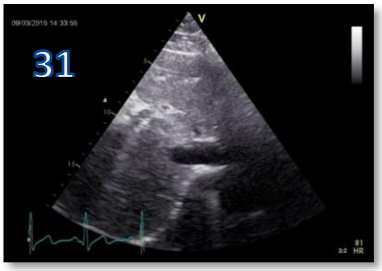
31

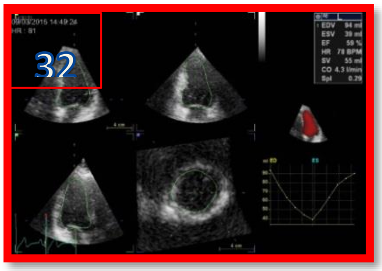
32

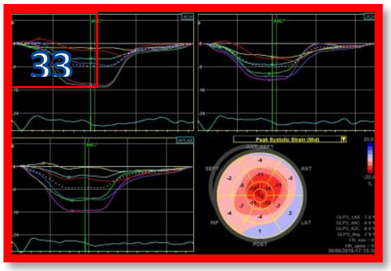
33
